# Spatiotemporal Features of the Three-Dimensional Architectural Landscape in Qingdao, China

**DOI:** 10.1371/journal.pone.0137853

**Published:** 2015-09-11

**Authors:** Peifeng Zhang

**Affiliations:** College of Pipeline and Civil Engineering, China University of Petroleum, Qingdao, Shandong, China; Chinese Academy of Sciences, CHINA

## Abstract

The evolution and development of the three-dimensional (3D) architectural landscape is the basis of proper urban planning, eco-environment construction and the improvement of environmental quality. This paper presents the spatiotemporal characteristics of the 3D architectural landscape of the Shinan and Shibei districts in Qingdao, China, based on buildings’ 3D information extracted from Quickbird images from 2003 to 2012, supported by Barista, landscape metrics and GIS. The results demonstrated that: (1) Shinan and Shibei districts expanded vertically and urban land use intensity increased noticeably from year to year. (2) Significant differences in the 3D architectural landscape existed among the western, central and eastern regions, and among the 26 sub-districts over the study period. The differentiation was consistent with the diverse development history, function and planning of the two districts. Finally, we found that population correlates positively with the variation in the 3D architectural landscape. This research provides an important reference for related studies, urban planning and eco-city construction.

## Introduction

Numerous urban areas have experienced continued horizontal expansions and sprawls through the decentralization process, while many cities expanded by increasing taller buildings as a response to land scarcity [1], due to the unprecedented urbanization since the launch of economic reforms in the late 1970s. Dramatic and irreversible land transformation has affected urban landscapes and environments both in and around cities. The characteristics of urban landscape are fundamentally important for urban design, planning and environmental studies. Recently, many studies have sought to reveal the spatiotemporal characteristics of urban landscape based on a time series of remotely sensed data [2–4], nighttime stable light data [5] and government statistics [6]. They were focus on the urban growth patterns [7], spatiotemporal dynamics of urban area growth [8, 9], urban land expansion rate [10], urban land use intensity [11] and the drivers of urban expansion [12, 13]. Generally, some important conclusions have been obtained. The built-up areas [8] and urban cover expanded rapidly, the urban spatial patterns was largely changed due to the urban expansion [9]. Extensive expansion was the main form of urban development [3]. The spatiotemporal dynamics of urban growth were closely associated with the economic conditions [10] and the development strategies [2, 4], etc. The above mentioned researches applied mature methods, contained original content and presented significant results to reveal the characteristics of urban expansion focused on urban sprawl, especially the horizontal expansion of urban land. While, due to land resource limitations, urban expansion occurs mainly in the vertical direction, through which building height and floor area ratio expand urban capacity, and urban landscapes change significantly as a result. Therefore, the study of two-dimensional urban landscape is unable to meet the needs of urban vertical expansion, and 3D urban landscape is a new trend.

Obviously, to understand the vertical expansion of urban areas requires urban expansion and landscape research, for which urban 3D information is vital. Unfortunately, quantitative urban 3D information is often unavailable, incomplete, or out-of-date. Information on original building plans is often poorly filed, stored and maintained, and it is extremely difficult to establish a full and accurate inventory of urban buildings and structures by piecemeal assembly of building design plans [14]. Field surveys are usually labor-intensive, time-consuming, error prone, and limited in their coverage. Various remote sensing images and methods have been used to efficiently extract urban building information, such as automatic 3D building reconstruction from aerial images [15–17], Lidar data [18–23] and synthetic aperture radar (SAR) images [24–26]. However, these methods also have deficiencies. Time series data of aerial images, Lidar data and SAR images are not always available because of discontinuous data collection periods and the high prices of the data itself, especially in developing countries. High level of professional knowledge is required for automatic 3D information extraction from these images because of complex image preprocessing and the establishment of regulation and reconstruction algorithms. Extraction accuracy cannot be guaranteed.

Fortunately, an alternative techniques for extracting 3D information from high resolution satellite imagery is via monoplotting [27, 28], a well-known photogrammetric technique for extracting 3D spatial information from aerial images of terrain described by a digital elevation model (DEM). Barista is a software package designed for extracting 3D information from high resolution satellite imagery via visual interpretation [28], and both RPC bundle adjustment and monoplotting functions have been included in the package. It has demonstrated that sub-pixel geo-positioning and 0.9 m height accuracy can be achieved with a single ground control point using the Rational Polynomial Coefficients (RPC, a sensor orientation model) bundle adjustment and monoplotting from IKONOS and QuickBird images [29–31]. The process requires a lower level of professional knowledge than alternative processes but still achieves good extraction accuracy. Accurate 3D information provides basic data for the research of urban vertical expansion and 3D landscapes. To date, few studies have examined spatiotemporal characteristics of urban 3D landscapes based on a time series of urban 3D information.

This study mainly explored the spatiotemporal characteristics of urban landscapes based on 3D building information extracted from Quickbird images (2003, 2006, 2009 and 2012) in Shinan and Shibei districts, Qingdao, China. We address two questions: (1) How to quantify 3D features of the urban architectural landscapes? (2) What are the spatiotemporal characteristics of 3D architectural landscapes in the study area from 2003 to 2012? Identifying the features of urban 3D architectural landscapes is important to guide urban planning, which is one of the main challenges to spatial planners in China.

## Study Area

Shinan and Shibei districts are both old areas of Qingdao, which is a rapidly developing and important eastern coastal city in Shandong, China. As a result of the socioeconomic development and urban renewal policy of Qingdao, the city has undergone gradual changes in both industrial structure and regional function. Shinan and Shibei districts expanded vertically with the increase of high-rise buildings from 2003 to 2012.

**Fig 1:**
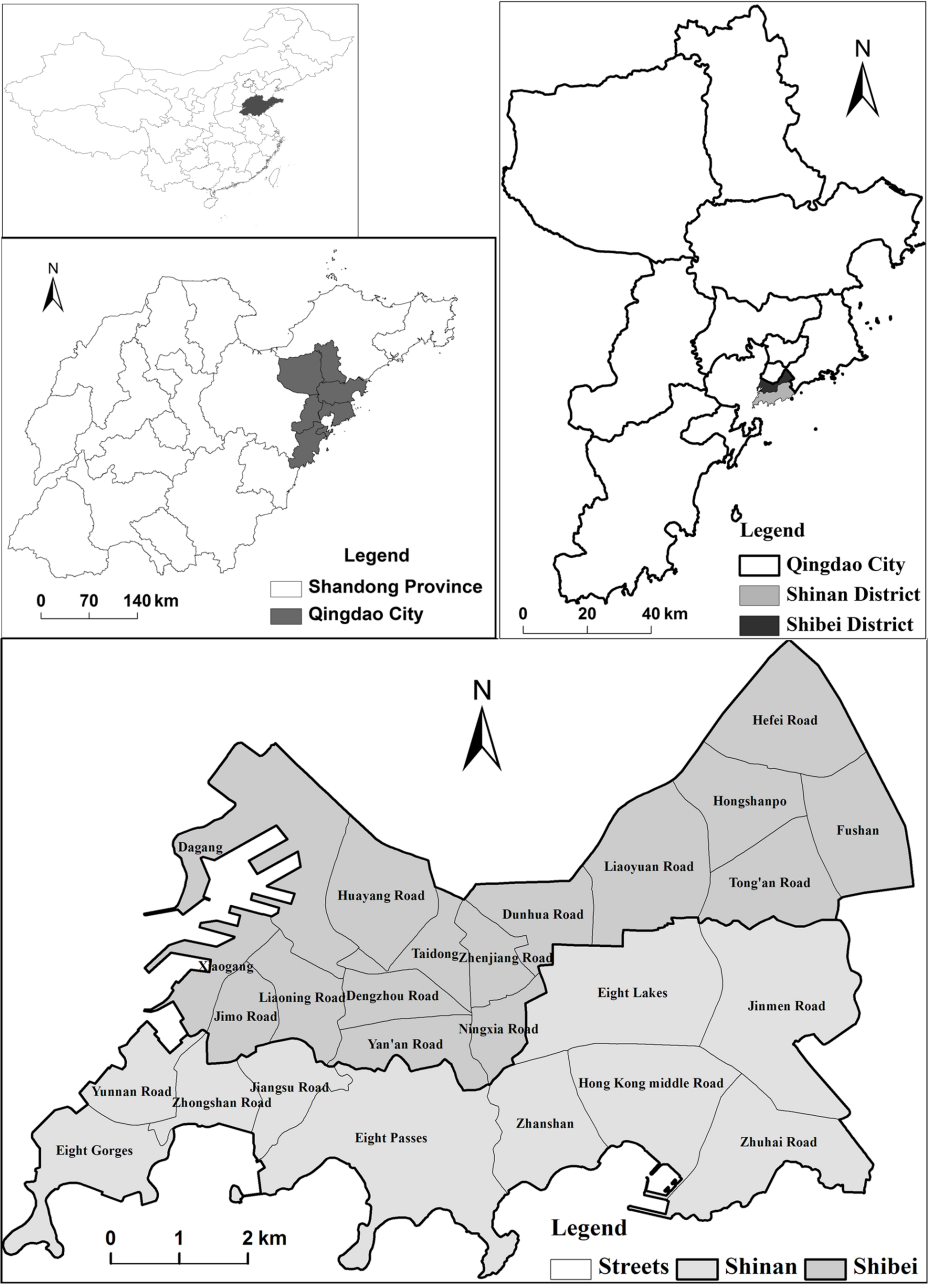
Location of the study area.

Shinan District is located in southern Qingdao, and has an area of 30.01 km^2^, with maximum dimensions of 12.7 km from east to west and 4.8 km from north to south (Fig 1), total population of 0.55 million in 2012. It is the current center of administration, tourism, culture, trade, finance and technology in Qingdao. As a multi-functional center, Shinan District comprises 10 sub-districts (Fig 1). These sub-districts perform different functions, namely business, tourism, service, administration, finance, or residential, and building types and shapes vary among them. Zhongshan Road Sub-district is the western business center of Qingdao. Eight Passes Sub-district is the cultural recreation area, and is noted for its European-style and urban conservation areas filled with traditional architecture, in a characteristic style that mixes Chinese and Western classical architecture. Hong Kong Middle Road Sub-district is the central area of the city and contains the eastern commercial district and modern services.

Shibei District has an area of 28.63 km^2^, and stretches 12.7 km from west to east and 4.8 km from north to south. It comprises 16 sub-districts (Fig 1). The central business district (CBD) of Qingdao is situated in the east part of the Dunhua Road Sub-district and the south part of the Liaoyuan Road Sub-district. Taidong Sub-district was the center of the Taidong Business Zone. Shibei District is an important industrial and commercial area, and most port and trade activity is located on the west coast of this district. In 2012, Shibei District had a population of 0.5 million and GDP of 50 billion RMB.

## Material and Methods

### Material

Primary data sources used in this study include QuickBird images with spatial resolution of 0.61m, acquired in February 2003, October 2006, September 2009 and January 2012 in the Shinan and Shibei districts of Qingdao. A 1:10000 scale Digital Elevation Model (DEM), and 22 Ground Control Points (GCPs) detected by GPS were used to extract buildings’ 3D information from the QuickBird images in Barista software [32]. Urban planning data and census data were obtained from the Qingdao Planning Bureau, Shinan and Shibei Statistical Yearbook.

### Indexes and Method

**Fig 2:**
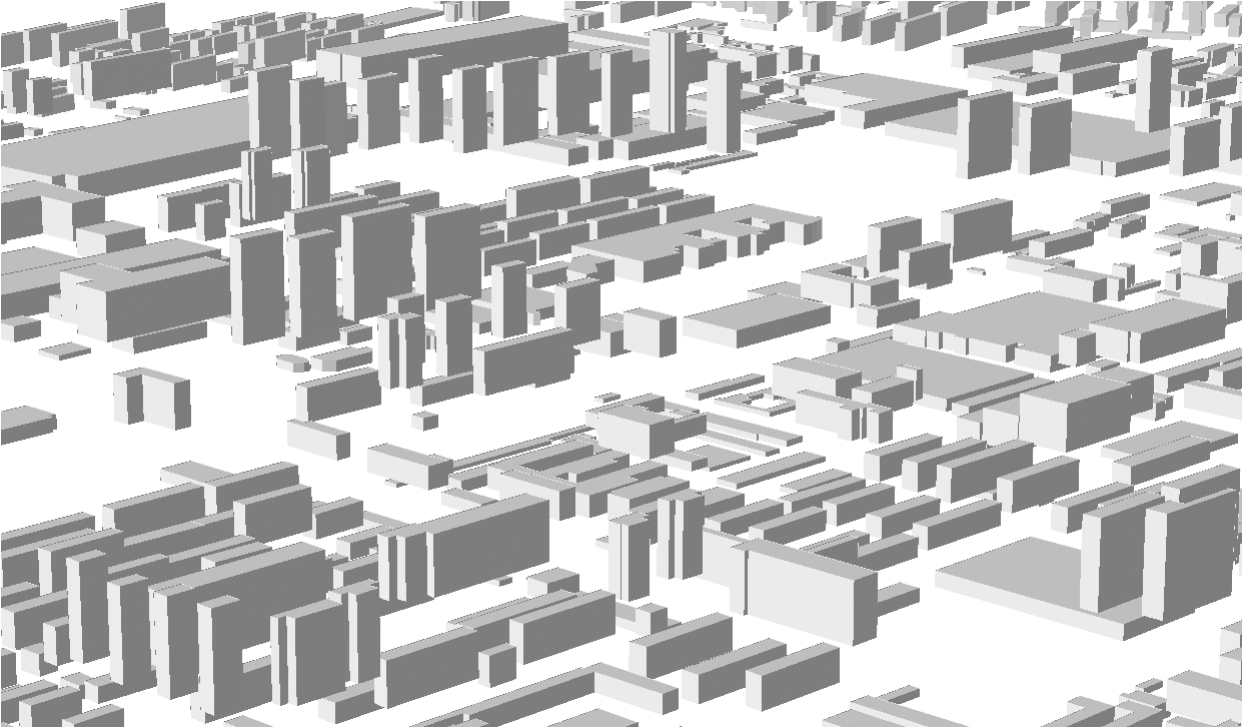
Extracted results of buildings’ three-dimensional information.

Buildings’ 3D information was extracted from the QuickBird images in Barista. Firstly, we obtained a set of bias-corrected RPCs based on RPB (the sensor orientation model for the QuickBird images) and the 22 GCPs using the RPC bundle adjustment function. Secondly, we measured a visible floor point of a building, then the roof point corresponding to that floor point and the remaining building roof boundary points in the same image, via monoplotting function (the monoplotter solves the planimetric position via least-squares estimation with the final height determined through interpolation from the DEM). Buildings’ 3D coordinates are determined by the intersection of the image ray with the vertical pole and horizontal plane passing through the floor point and the first roof point, respectively. And both the buildings’ wireframe and height (Fig 2) were obtained. Then, most data were processed using ArcGIS. To validate the accuracy of the extracted building height, we randomly measured 581 buildings in the field used to calculate the height accuracy and a 91.8% accuracy rate was achieved.

Developing 3D cities requires an improved evaluation indicator for urban landscape studies. In this article, we established ten indexes including number of buildings (N) [32], average area (AV_A), average height (AV_H), standard deviation of height (SD_H), building coverage ratio (BCR), floor area ratio (FAR), standard deviation of area (SD_A), standard deviation of volume (SD_V) [1], land use intensity (LUI) and population density (P_D) (Table 1), which reflect the architectural landscape from the aspects of fragmentation degree, height, morphology, density, degree of space congestion and land use intensity. These indexes were used to estimate the spatiotemporal characteristics of 3D urban architectural landscapes in the study area from 2003 to 2012.

**Table 1:** Index of terms and their meanings.

Attribution	Expression	Description
Number of buildings (*N*)	*N* = *n*	Number of buildings in a district. Describe the fragmentation degree of the architectural landscape. N>0.
Average Area (*AV_A*)	$AV\_A=\frac{\sum_{i=1}^{n}A_{i}}{n}$	The average area of buildings in a district. Describe the fragmentation degree of the architectural landscape. AV_A>0 (m^2^).
Average Height (*AV_H*)	$AV\_H=\frac{\sum_{i=1}^{n}H_{i}}{n}$	The average value of buildings’ height in a district. AV_H>0 (m).
Standard deviation of Height (*SD_H*)	$SD\_H=\sqrt{\frac{\sum_{i=1}^{n}{\left(H_{i}-AV\_H\right)}^{2}}{n}}$	Reflect the degree of dispersion and variation of building height, the smaller standard deviation the smaller variation. SD_H≥0.
Building Coverage Ratio (*BCR*)	$BCR=\frac{\sum_{i=1}^{n}F_{\;i}}{A}$	Reflect the density of buildings and congestion degree in the horizontal direction. 0≤BCR≤1.
Floor Area Ratio (*FAR*)	$FAR=\frac{\sum_{i=1}^{n}\left(H_{i}/C^{\;*}\;F_{i}\right)}{A}$	Reflect the density and accommodating capability of urban area in three-dimensional space. FAR≥0.
Standard deviation of Area(*SD_A*)	$SD\_A=\sqrt{\frac{\sum_{i=1}^{m}{\left(A_{i}-\mathrm{AV}\_A\right)}^{2}}{\text{n}}}$	Reflect the degree of dispersion and variation of building area, the smaller standard deviation the smaller variation. SD_A≥0.
Standard deviation of Volume (*SD_V*)	$SD\_V=\sqrt{\frac{\sum_{i=1}^{m}{\left(V_{i}-\mathrm{AV}\_V\right)}^{2}}{\text{n}}}$	Reflect the degree of dispersion and variation of building volume, the smaller standard deviation the smaller variation. SD_V≥0.
Land Use Intensity(LUI)	$LUI=\frac{\sum_{i=1}^{n}V_{\;i}}{A}$	Reflect the intensity of land use in urban district. The higher of LUI the greater efficiency of land use. LUI≥0.
Population Density(*P_D*)	$P\_D=\frac{P}{A}$	Reflect the number of population on per unit area. *P_D*>0(*10^4^/km^2^).

*H*_*i*_, *A*_*i*_, *V*_*i*_ and *F*_*i*_ are the height, area, volume and floor area of the *i*th building in a district, respectively; *n*, *m* and *P* are the numbers of buildings, blocks and population in a district, respectively; *A* is the total area of the district, and *C* is a constant (C = 3.0 m).

## Results

### Characteristics of architectural landscapes

**Fig 3:**
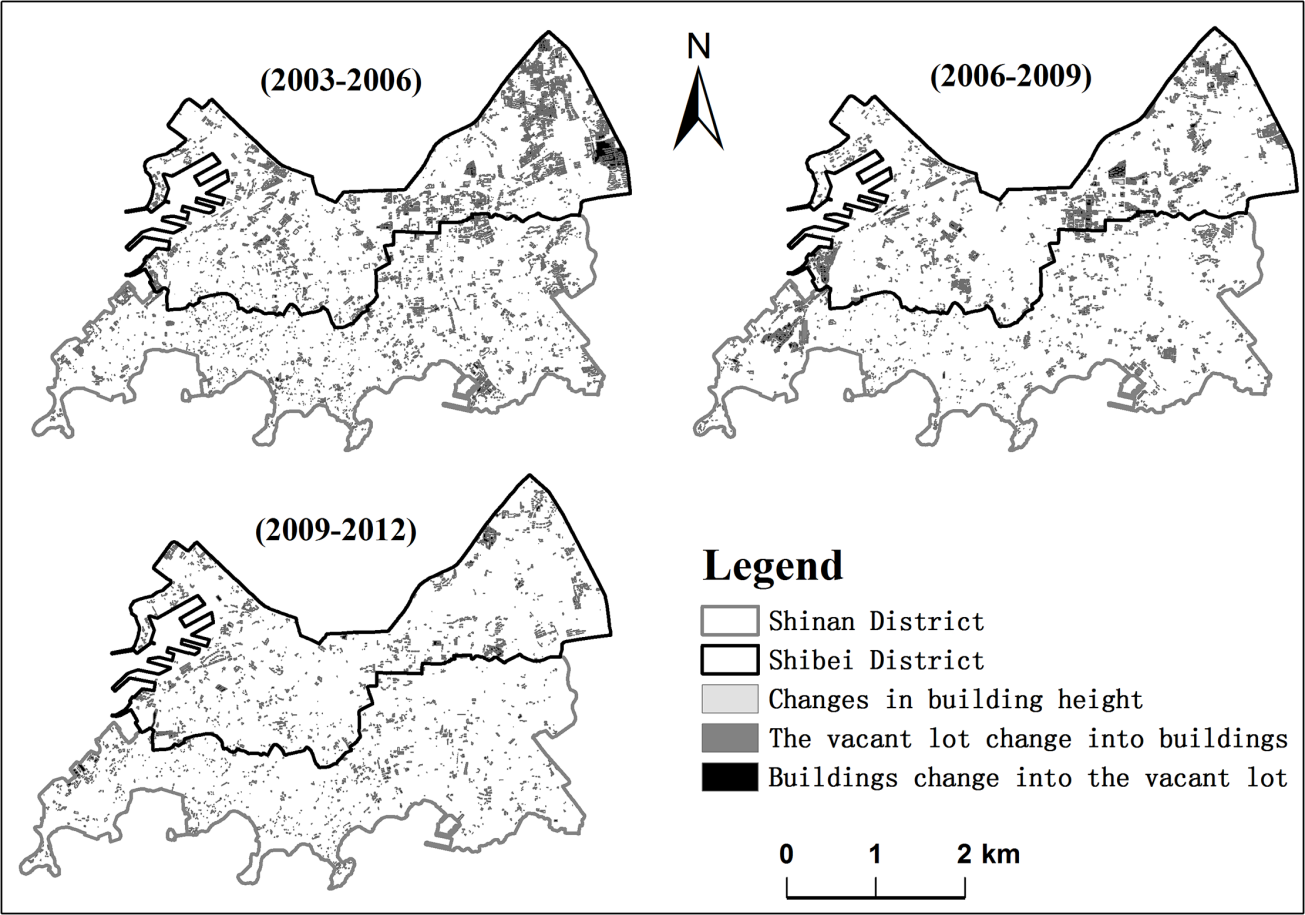
Spatiotemporal distribution of the changed buildings from 2003 to 2012.

The architectural landscapes, especially the spatiotemporal distribution and building height changed significantly in the Shinan and Shibei districts from 2003 to 2012 (Fig 3). A comparison of results from the four years of data (Table 2) shows a gradual change in the 3D architectural landscape in the study area. The standard deviation of height (SD_H), average height (AV_H) and floor area ratio (FAR) increase from 11.86 m to 16.66 m, 15.35 m to 20.35 m and 1.3 to 1.62 in Shinan District, and from 12.58 m to 17.42 m, 17.56 m to 26.11 m and 1.64 to 2.13 in Shibei District, respectively. The number of buildings (N) and the building coverage ratio (BCR) decrease from 11186 to 9532 and 0.2 to 0.18 in Shinan and from 8855 to 7938 and 0.24 to 0.21 in Shibei, respectively. The average area (AV_A) increases in Shinan but decreases in Shibei from 2003 to 2012. The variation indicating the fragmentation of the architectural landscape reduces, while the differences in building height and land use intensity of the study area increase from 2003 to 2012. Simultaneously, the urban landscape expands vertically and open space among buildings enlarges horizontally. According to our analysis, the demands for a higher quality urban environment, increased urban population and scarce land resources are the main impetus for the variation of the 3D urban architectural landscape.

**Table 2:** Results of the architectural landscape in the study area.

Year	N		AV_A (m^2^)		SD_H(m)		AV_H (m)		BCR		FAR	
	Shinan	Shibei	Shinan	Shibei	Shinan	Shibei	Shinan	Shibei	Shinan	Shibei	Shinan	Shibei
2003	11186	8855	524.22	773.66	11.86	12.58	15.35	17.56	0.20	0.24	1.30	1.64
2006	10050	7826	565.03	828.27	13.96	14.24	17.59	21.39	0.19	0.23	1.42	1.76
2009	10022	7728	564.10	778.34	15.87	16.79	18.97	24.50	0.19	0.21	1.57	1.96
2012	9532	7938	573.44	760.59	16.66	17.42	20.35	26.11	0.18	0.21	1.62	2.13

Another important piece of information in Table 2 is the significant differences in 3D architectural landscapes between Shinan and Shibei districts. Shibei has higher AV_A, SD_H, AV_H, BCR and FAR than Shinan, as well as having fewer N, suggesting its higher land use intensity and lower fragmentation of the architectural landscape. The variation and regional differences are an inevitable result of the decreased availability of land resources in the urbanizing process and differentiation of urban planning and regional development.

### Differences in architectural landscapes among streets

**Fig 4:**
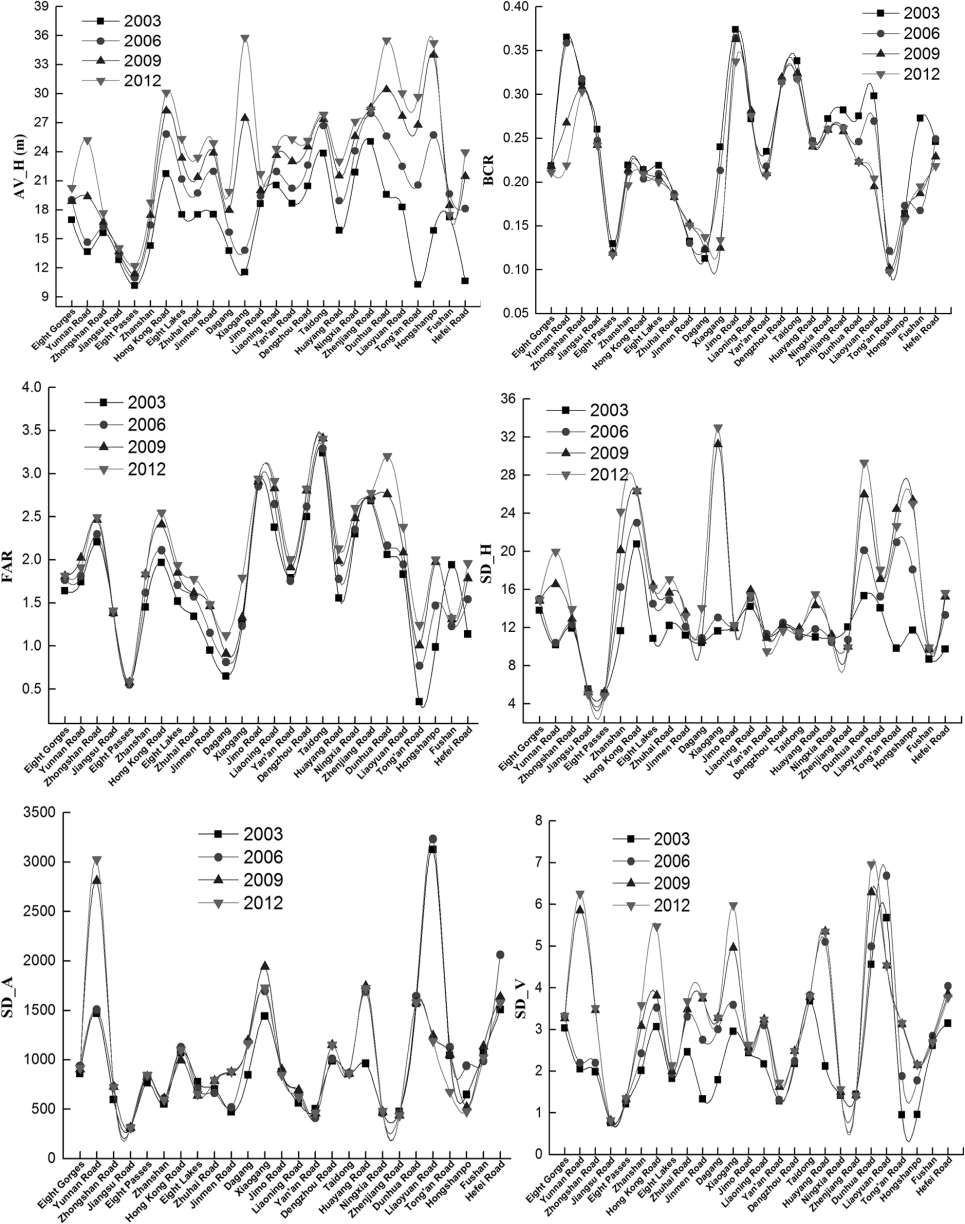
Variation of 3D architectural landscapes among streets.

Fig 4 shows the variation and spatiotemporal differences of 3D urban architectural landscapes among 26 sub-districts in the study area from 2003 to 2012. The value of average height (AV_H) and floor area ratio (FAR) gradually increases but the building coverage ratio (BCR) gradually decreases in 26 streets, indicating that all sub-districts improve land use efficiency by increasing building height during urbanization, and the urban area expanded vertically instead of sprawling horizontally. Both standard deviation of height (SD_H) and volume (SD_V) increase yearly in most streets, and for most streets the standard deviation of area (SD_A) remains substantially unchanged except for Yunnan Road (an increase) and Liaoyuan Road (a decrease). This characteristic suggests the dispersion and variation of building height and volume increase yearly, and building area changes slightly in most streets.

The calculation results from the 26 sub-districts suggest obvious spatial heterogeneity of the urban architectural landscapes across the western (Eight Gorges, Yunnan Road, Zhongshan Road, Jiangsu Road, Liaoning Road, Jimo Road, Xiaogang and Dagang), central (Eight Passes, Zhanshan, Yan'an Road, Ningxia Road, Dengzhou Road, Huayang Road, Taidong, Zhenjiang Road and Dunhua Road) and eastern (Hong Kong Middle Road, Eight Lakes, Jinmen Road and Zhuhai Road, Liaoyuan Road, Tong'an Road, Hongshanpo, Fushan and Hefei Road) regions of the study area since 2003 (Fig 1 and Fig 4). The values of these indexes differ significantly among the western (W), central (C) and eastern (E) regions. The rankings are W>C>E for BCR, SD_H and SD_V, E>C>W for AV_H, C>W>E for FAR and W>E>C for SD_A. The western region has the largest variation in building shapes (height, area and volume), the smallest area of open space among buildings and the lowest average building height. The eastern region has the smallest variation in building shapes, the lowest land use intensity and the highest average building height. The central region has the highest land use intensity and the smallest variation in building area.

This differentiation primarily resulted from differences among the regions in development history, function and planning. Qingdao originated from a fishing village on the western coast of the peninsula, and then expanded to the north and east. Buildings in the west of the city are mostly low-rise and multi-story, those in the central region are predominantly multi-story and high-rise, and those in the east are high-rise. Since 1994, the center of urban development has shifted from the west (the Zhongshan Road Sub-district) to the east (the Hong Kong Middle Road Sub-district). Meanwhile, the urban renewal process in the west saw most low-rise buildings replaced by high-rise buildings. In recent years, Taidong, the east part of the Dunhua Road Sub-district and the west part of the Liaoyuan Road Sub-district became the central business district (CBD) of Qingdao. The eastern region is a newly developed area in which some low-rise buildings still exist, while the central region is reaching maturity and most of its buildings are multi-story and high-rise.

Considerable fluctuation emerges for all indexes among the 26 sub-districts (Fig 4). The Xiaogang, Dunhua Road, Hongshanpo, Liaoyuan Road, Ningxia Road, Jimo Road, Yunnan Road, Taidong, Dengzhou Road and Zhongshan Road sub-districts occupy the peak for AV_H, BCR and FAR, while the Dagang, Eight Passes and Tong'an Road sub-districts occupy the valley for these same values. The Xiaogang, Hong Kong Middle Road, Hongshanpo, Zhanshan, Tong'an Road, Liaoyuan Road, Yunnan Road, Huayang Road and Dunhua Road sub-districts occupy the peak for SD_A, SD_V, SD_H, while Jiangsu Road, Eight Passes etc. occupy the valley. In 2012, the highest and lowest values for AV-H were in Xiaogang (35.79 m) and Eight Passes (12.21 m), those for BCR were in Jimo Road (0.34) and Tong'an Road (0.1), those for FAR were in Taidong (3.4) and Eight Passes (0.59), those for SD_H were in Xiaogang (33.02 m) and Eight Passes (4.84 m), those for SD_A were in Yunnan Road (3027.06 m^2^) and Jiangsu Road (314.13 m^2^), and those for SD_V were in the Dunhua Road (6.95 m^3^) and Jiangsu Road (0.82 m^3^) sub-districts, respectively. This illustrates significant differentiation of 3D urban architectural landscapes among the sub-districts over the study period, and that Eight Passes has the lowest values for nearly all the indexes.

## Discussion and Conclusion

In this article, monoplotting proves extraordinarily effective in collecting highly accurate building 3D information. However, this method has some limitations. Errors in building height are inevitable because the accuracy of monoplotting depends on the quality of the DEM, the sensor orientation model, the image measurement precision, and the off-nadir angle of the satellite image [33]. The monoplotting process is time-consuming owing to the need to extract data through visual interpretation. There is a greater requirement for data quality, because at least one building point at ground level and the corresponding roof point can be measured, the measurement of buildings from an individual high resolution satellite image is possible. We believe this method would be useful for urban planning and community management to characterize and quantify urban spatial structure based on high resolution satellite images. The integrated application of 3D architectural information from Quickbird images and landscape indexes represents the spatiotemporal variation of the 3D urban architectural landscape in the study area.

### Spatiotemporal variation of land use intensity

**Fig 5:**
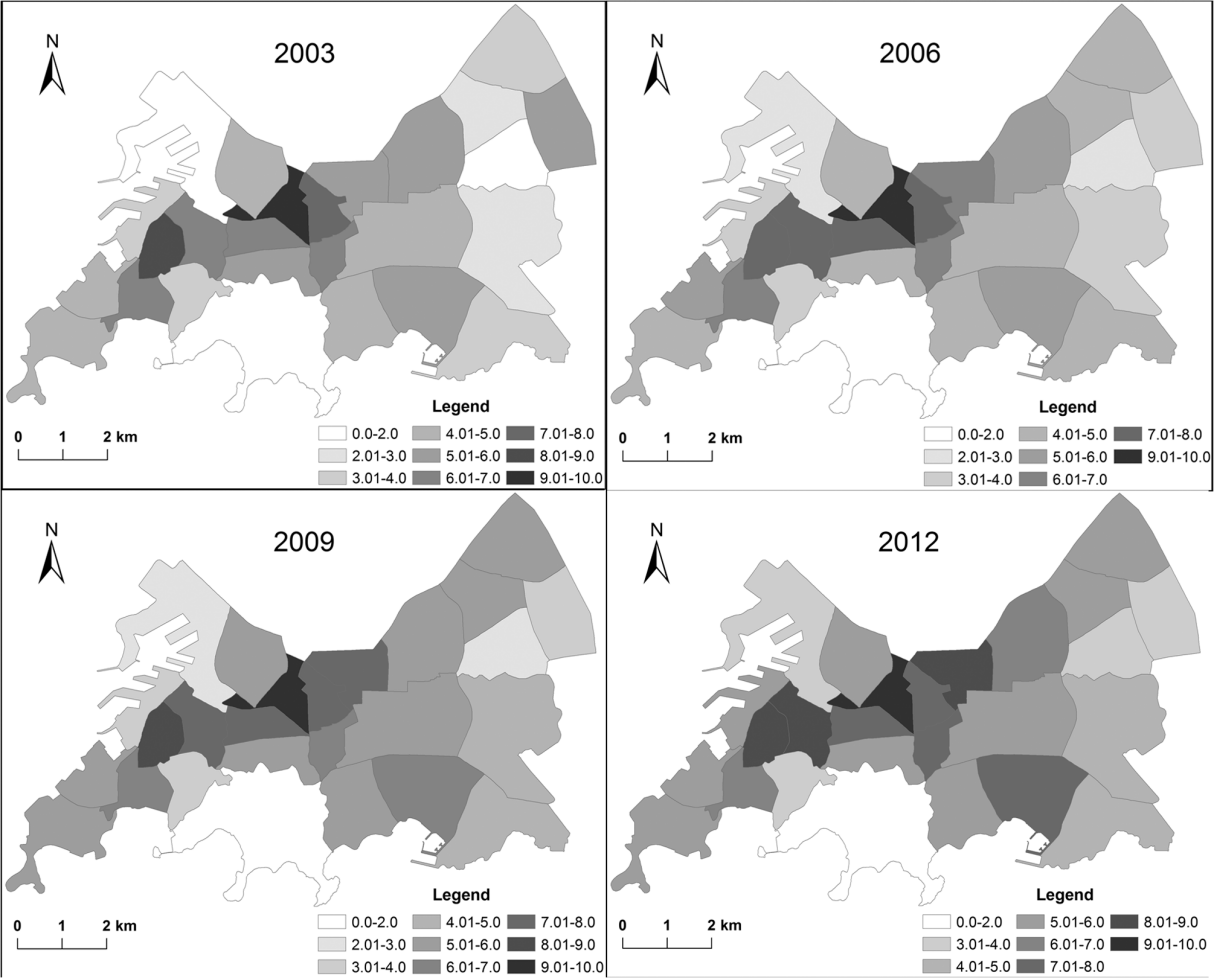
Characteristics of land use intensity (LUI) in the study area.

Significant variation and differentiation of the urban architectural landscape over the past 10 years has been demonstrated in the study area and at the sub-district level. Fig 5 is the classification maps of LUI over the study period. The yearly increase in LUI in the 25 sub-districts other than Eight Passes was less than 2 from 2003 to 2012. And obvious differences existed among the 26 sub-districts. The Taidong and Jimo Road sub-districts best embodied the vertical extension of urban growth, with extremely high LUI. Over time, LUI in the Zhongshan Road, Liaoning Road, Dengzhou Road, Dunhua Road and Liaoyuan Road sub-districts increased significantly and the increase clearly exceeded that in other sub-districts. LUI in the Hong Kong Middle Road Sub-district also increased rapidly and clearly exceeded that in the surrounding sub-districts from 2003 to 2012. In contrast, the Eight Passes Sub-district has the lowest LUI and slow urban vertical growth.

Fig 5 further demonstrates the spatiotemporal characteristics (Table 2, Fig 4) of the 3D architectural landscape in the study area during the research period. The urban LUI increased yearly and exhibited clear differentiation among sub-districts. The famous business districts (Zhongshan Road, Jimo Road, Taidong, Dunhua Road, Liaoyuan Road and Hong Kong Middle Road) have higher LUI than the other districts. And LUI increased rapidly around the business district. This indicated that high LUI was closely related to business function, and the growth rate of LUI increased with proximity to the business district.

### Factors impacting on the spatiotemporal change of the urban 3D architectural landscape

Urbanization is a major cause of spatiotemporal characteristics of the urban architectural landscape and significantly influences the structure and function of urban land use. It is a complex process involving numerous factors such as topography, economy and population growth, resident income, urban infrastructure construction, urban planning and policy [34], but is generally determined by population and economy. However, population is the main driver of economic development. Population growth increases demand for residences, transportation, public facilities, etc. which means more land resources, urban space and economic development are needed to meet this demand. Population is fundamental to urbanization and change in the urban landscape.

Table 3 shows the relationship of population density and 3D architectural landscape indexes (average building height, building coverage ratio and floor area ratio). Population density is positively correlated with AV-H, BCR and FAR at both the 5% and 1% significance levels. This indicates that high population density promotes increases in building height and urban land use efficiency to meet the demands of urban development.

**Table 3:** Correlation between population and architectural landscape.

Index		AV-H	BCR	FAR
P_D (*10^4^/km^2^)	Correlation coefficient	0.248*	0.504**	0.513**
	Significant	0.011	0.000	0.000

** P <0.01

* P <0.05.

### Significance of the 3D architectural landscape

Urban vertical development is the main trend of urbanization owing to the shortage of urban land resources. The characteristics of the 3D architectural landscape are both results and reflections of urbanization in China. The sustainable development of the urban structure and layout is the basis of urban planning, eco-environment construction and the improvement of environmental quality. Research on the spatiotemporal characteristics of the 3D architectural landscape in Qingdao reveals the true features of urbanization in just one part of China and has had considerable socioeconomic benefits, such as providing references for both similar research and proper urban planning, reducing urban environmental pollution and promoting urban economic development. Also, analysis of the 3D architectural landscape is becoming an important basis for improving environmental quality, promoting coordinated development of urban populations and urban environments and judging the ecological sustainability of urban areas. Such analysis can provide efficient reference tools to quantitatively evaluate and compare the impact of alternative plans and designs to enable urban planners and designers to make more informed development choices.

Spatiotemporal characteristics of urban 3D architectural landscape provide an important basis for analyzing the ecological and socioeconomic functions of urban spatial structure and are fundamental to the intelligent management and planning of urban environments. Different districts have distinct urban economic, social and cultural functions, and features such as building size, height, volume, density, morphology and vertical roughness vary among districts, so understanding the diversities of the urban 3D architectural landscape can help us to assess and create future planning scenarios.

## Supporting Information

S1 Table Data of AV-H, BCR, FAR and P_D in the 26 sub-districts from 2003 to 2012. (XLSX)
